# Tegumentary leishmaniasis and coinfections other than HIV

**DOI:** 10.1371/journal.pntd.0006125

**Published:** 2018-03-01

**Authors:** Dalila Y. Martínez, Kristien Verdonck, Paul M. Kaye, Vanessa Adaui, Katja Polman, Alejandro Llanos-Cuentas, Jean-Claude Dujardin, Marleen Boelaert

**Affiliations:** 1 Instituto de Medicina Tropical Alexander von Humboldt, Universidad Peruana Cayetano Heredia, Lima, Peru; 2 Department of Public Health, Institute of Tropical Medicine Antwerp, Antwerp, Belgium; 3 Faculty of Medicine and Health Sciences, University of Antwerp, Antwerp, Belgium; 4 Centre for Immunology and Infection, Department of Biology and Hull York Medical School, University of York, York, United Kingdom; 5 Department of Biomedical Sciences, Institute of Tropical Medicine Antwerp, Antwerp, Belgium; 6 Department of Biomedical Sciences, Faculty of Pharmaceutical, Biomedical and Veterinary Sciences, University of Antwerp, Antwerp, Belgium; Instituto de Ciências Biológicas, Universidade Federal de Minas Gerais, BRAZIL

## Abstract

**Background:**

Tegumentary leishmaniasis (TL) is a disease of skin and/or mucosal tissues caused by *Leishmania* parasites. TL patients may concurrently carry other pathogens, which may influence the clinical outcome of TL.

**Methodology and principal findings:**

This review focuses on the frequency of TL coinfections in human populations, interactions between *Leishmania* and other pathogens in animal models and human subjects, and implications of TL coinfections for clinical practice. For the purpose of this review, TL is defined as all forms of cutaneous (localised, disseminated, or diffuse) and mucocutaneous leishmaniasis. Human immunodeficiency virus (HIV) coinfection, superinfection with skin bacteria, and skin manifestations of visceral leishmaniasis are not included. We searched MEDLINE and other databases and included 73 records: 21 experimental studies in animals and 52 studies about human subjects (mainly cross-sectional and case studies). Several reports describe the frequency of *Trypanosoma cruzi* coinfection in TL patients in Argentina (about 41%) and the frequency of helminthiasis in TL patients in Brazil (15% to 88%). Different hypotheses have been explored about mechanisms of interaction between different microorganisms, but no clear answers emerge. Such interactions may involve innate immunity coupled with regulatory networks that affect quality and quantity of acquired immune responses. Diagnostic problems may occur when concurrent infections cause similar lesions (e.g., TL and leprosy), when different pathogens are present in the same lesions (e.g., *Leishmania* and *Sporothrix schenckii*), or when similarities between phylogenetically close pathogens affect accuracy of diagnostic tests (e.g., serology for leishmaniasis and Chagas disease). Some coinfections (e.g., helminthiasis) appear to reduce the effectiveness of antileishmanial treatment, and drug combinations may cause cumulative adverse effects.

**Conclusions and significance:**

In patients with TL, coinfection is frequent, it can lead to diagnostic errors and delays, and it can influence the effectiveness and safety of treatment. More research is needed to unravel how coinfections interfere with the pathogenesis of TL.

## Author summary

Infectious diseases are often studied one by one, but people can have more than one infection at the same time. This is likely to happen when different microorganisms are linked to specific geographical regions or living conditions. In this paper, we summarise the literature about infections occurring together with tegumentary leishmaniasis (TL), a disease of skin and mucosal tissues that is caused by *Leishmania* parasites. We found that in Latin America, patients with TL are often also infected with helminths or with *Trypanosoma cruzi* (the parasite that causes Chagas disease). Information from other parts of the world is scarce. Animal studies and observations in humans show that one infection can change the course of another infection, but how this happens is not well understood. When different infections affect the same patient at the same time, the diagnosis can be difficult, especially when different microorganisms are biologically similar, when they cause similar lesions, or when they are present in the same lesions. Treatment can also be difficult because some coinfections reduce the efficacy of the treatment against *Leishmania* and because some drug combinations can lead to cumulative adverse effects.

## Introduction

Tegumentary leishmaniasis (TL) is a disease of the skin and mucosal tissues caused by several species of the genus *Leishmania* (Protozoa, Trypanosomatida, Trypanosomatidae) that are transmitted by the bite of phlebotomine sandflies [1]. Parasites belonging to the subgenus *Leishmania* are found in the Old and the New World, whereas those of the subgenus *Viannia* are restricted to the New World [1–3]. *Leishmania* parasites produce a wide spectrum of clinical manifestations in humans and other mammals, ranging from asymptomatic infection to life-threatening disease [1–3]. Yearly, an estimated 1 million people develop TL, mainly in Bolivia, Brazil, Colombia, Peru, Algeria, Tunisia, Saudi Arabia, Syria, Iran, Afghanistan, and Pakistan [4].

The overlapping geographical distribution of TL with many highly prevalent (e.g., helminthiasis) [5] and some less common (e.g., leprosy) [6] infectious diseases, as well as experimental studies [7], together indicate the importance of understanding how coinfections may alter the outcome of TL and vice versa. Indeed, several infectious diseases linked to poverty, housing conditions, hygiene, or to vectors that thrive in similar circumstances tend to affect the same populations [8–12]. It is therefore likely that in the tropical and temperate regions where TL occurs, many people carry more than one pathogen at once, although the epidemiology of such coinfections is not well known. Furthermore, the clinical outcome of *Leishmania* infection depends on characteristics of both the *Leishmania* parasite and the human host immune response [13–16]. Pathogens other than *Leishmania* may modulate this host immune response and consequently influence the natural history of TL as well as the response to antileishmanial treatment [12,16].

The most frequently studied coinfection is that between *Leishmania* and human immunodeficiency virus (HIV), in that the natural history of each of the two infections is modified by the presence of the other [17]. HIV increases the risk of severe and disseminated TL, and some HIV-infected patients develop visceral leishmaniasis in the presence of *Leishmania* species that are usually only dermotropic [17–19]. HIV also increases the risk of TL recurrence and treatment failure [18,19]. On the other hand, leishmaniasis interferes with monocyte and macrophage function in such a way that it facilitates HIV progression [20]. Interactions between TL and infections other than HIV have not been comprehensively reviewed before.

The objectives of the present review are to summarise the evidence about the (i) frequency of TL and coinfections other than HIV in human populations, (ii) interactions between *Leishmania* and other pathogens in animal models and human subjects, and (iii) implications of TL coinfections for clinical practice.

## Methods

### Eligibility criteria

We searched the medical literature to identify publications about TL and coinfections. For the purpose of this review, we defined TL as all forms of cutaneous (localised, disseminated, or diffuse) and mucocutaneous leishmaniasis. Records about the skin manifestations caused by *L*. *donovani* and *L*. *infantum/L*. *chagasi* (such as post–kala-azar dermal leishmaniasis) were not included because the main clinical outcome of these infections is visceral leishmaniasis, which is outside the scope of this review.

Records about HIV/AIDS and TL were not included because this topic has already been extensively reviewed elsewhere [17–19]. Records about the contamination or superinfection of TL lesions with gram-positive or gram-negative bacteria of the skin such as *Staphylococcus aureus* or *Streptococcus pyogenes* were also excluded. Review papers were not included. We did not restrict the search by geographical region, study design, language of publication, or publication date.

### Information sources and search

The databases MEDLINE, Embase, LILACS, Scielo, Cochrane, and African Index Medicus as well as local library databases, searched in August 2017, were the information sources for this review. We used search terms indicating (groups of) infections, pathogens, and diseases caused by these pathogens. The detailed search strategy for MEDLINE is given in S1 File. We also reviewed the reference lists of selected articles.

### Data collection and synthesis

Two reviewers extracted the data from the included records; any doubts and discordances were resolved through discussion. Specific points of interest while reading and summarising the articles were (i) frequency of coinfection in humans, (ii) mechanisms of interaction and effect of coinfection on TL progression, and (iii) potential implications for clinical management. We described the information the same way the authors of the original publications did, using mainly counts, proportions, and medians.

We used the Preferred Reporting Items for Systematic Reviews and Meta-Analyses (PRISMA) statement [21] to prepare this review, but it was not possible to follow all the recommendations because PRISMA mainly focuses on the evaluation of healthcare interventions and our focus was broader than that. The PRISMA checklist is given in S1 List.

## Results

### Study selection and characteristics

**Fig 1:**
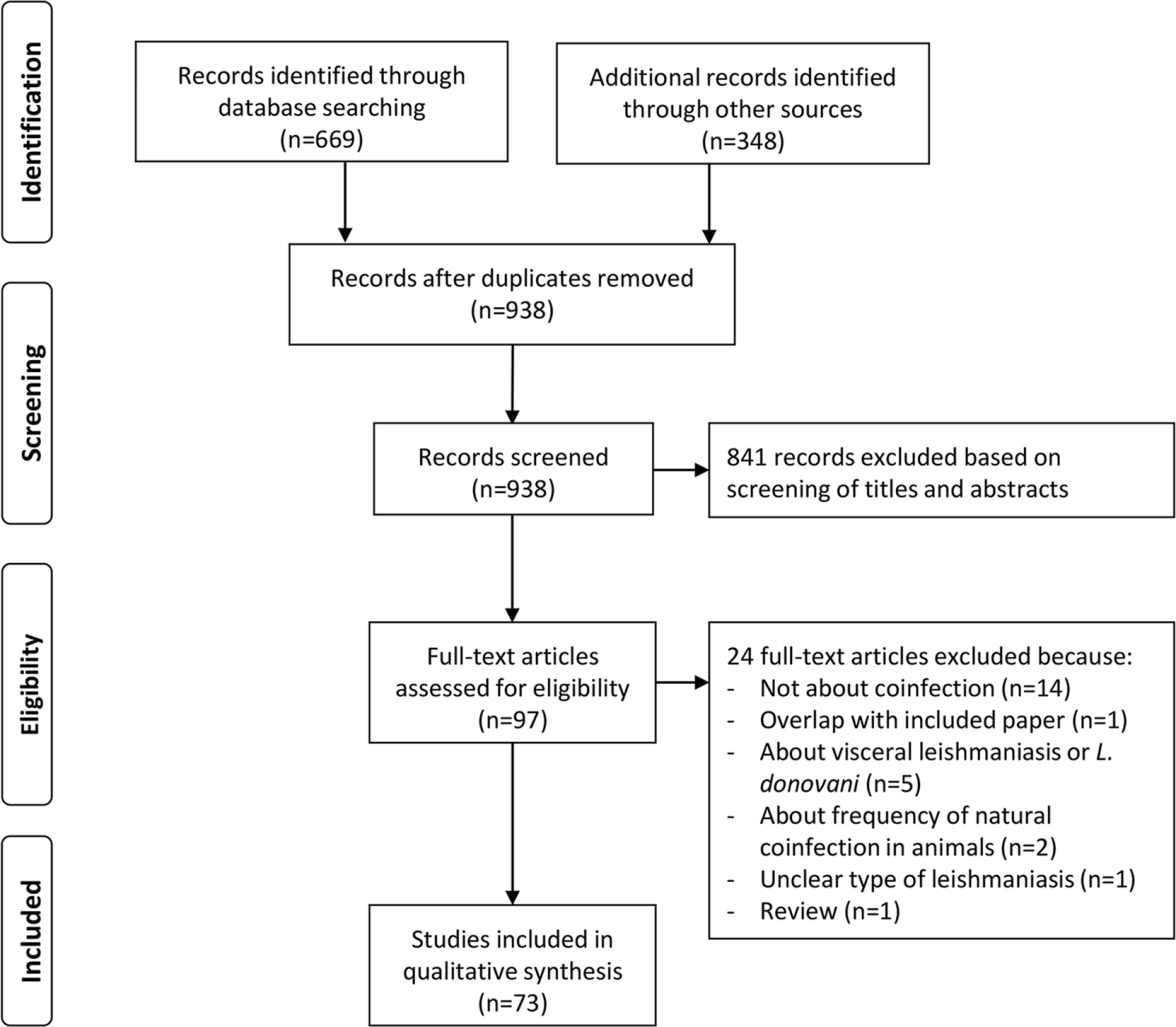
Flow diagram of record search and selection.

The MEDLINE search retrieved 669 records, and searching other databases yielded 348 additional records. After reading titles or abstracts or both, we removed 79 duplicates and discarded 841 records because they were not relevant (Fig 1). The most frequent reason for dropping records was that, while leishmaniasis and another infection were mentioned in the same text, the publication was not about coinfection (e.g., a paper about different infections occurring in the same region but not affecting the same persons). We assessed the remaining 97 full-text records for eligibility and retained 73 for the present review (Fig 1).

The 73 articles included in this review had different study designs (Table 1). There were 21 original research papers about experimental studies of coinfection in animal models and 52 original research papers about coinfection in human patients. The 52 studies about human subjects included 1 clinical trial, 2 cohort studies, 13 cross-sectional or prevalence studies, 7 studies on the development or performance of diagnostic tests, 24 case series or case reports with a clinical focus, and 5 case series or reports with an immunological focus. The coinfecting pathogens for which we found the highest number of records were *Trypanosoma cruzi* (*n* = 18), *Mycobacterium leprae* (*n* = 14), helminths (*n* = 12), and *M*. *tuberculosis* (*n* = 9). Two records addressed coinfection of *Leishmania* with more than one pathogen (Table 1).

**Table 1:** Overview of all studies about TL and coinfections included in this review.

Coinfecting pathogen	Study design	Number of studies	Number of human cases with coinfection	References to included studies
**Helminths**				
*Ancylostoma duodenale*, *Ascaris lumbricoides*, *Schistosoma mansoni*, *Strongyloides stercoralis*, and/or *Trichuris trichiura*	Randomised clinical trial	1	90	[22]
*A*. *duodenale*, *A*. *lumbricoides*, *S*. *mansoni*, *S*. *stercoralis*, and/or *T*. *trichiura*	Cohort study	2	122	[5,12]
*Litomosoides sigmodontis*, *Nippostrongylus braziliensis*, *S*. *mansoni*, *Strongyloides ratti*, or *Taenia crassiceps*	Experimental study in animals	8	Not applicable	[7,23–29]
**Protozoa**				
*Trypanosoma cruzi*	Cross-sectional study in general population	1	11	[30]
*T*. *cruzi*	Cross-sectional study in TL patients^a^	7	211^a^	[31–37]
*T*. *cruzi*	Study about diagnostic tests^a^	6	74^a^	[38–43]
*T*. *cruzi*	Immunological study in humans	1	16	[44]
*T*. *cruzi*	Case report/series	1	1	[45]
*T*. *cruzi*	Experimental study in animals	2	Not applicable	[46,47]
*Trypanosoma brucei*	Experimental study in animals	2	Not applicable	[48,49]
*Toxoplasma gondii*	Cross-sectional study in TL patients	1	2	[37]
*T*. *gondii*	Immunological study in humans	1	16	[50]
*T*. *gondii*	Experimental study in animals	2	Not applicable	[51,52]
*Plasmodium* sp.	Experimental study in animals	7	Not applicable	[53–59]
**Fungi**				
*Sporothrix schenckii*	Case report/series	2	4	[60,61]
*S*. *schenckii*	Study about diagnostic tests	1	0	[62]
*Paracoccidioides braziliensis*	Cross-sectional study in TL patients	1	2	[37]
*P*. *braziliensis*	Cross-sectional study in patients with paracoccidioidomycosis	1	10	[63]
*Coccidioides posadasii*	Cross-sectional study in TL patients	1	1	[37]
*Cryptococcus laurentii*	Case report/series	1	1	[64]
**Mycobacteria**				
*Mycobacterium tuberculosis*	Cross-sectional study in TL patients	1	3	[37]
*M*. *tuberculosis*	Case report/series	8	9	[65–72]
*Mycobacterium leprae*	Case report/series	12	25	[6,70,73–82]
*M*. *leprae*	Case report/series of leprosy patients immunised with live *Leishmania tropica*	2	0	[83,84]
*Mycobacterium ulcerans*	Case report/series	1	1	[85]
**Other bacteria**				
*Treponema pallidum*	Cross-sectional study in TL patients	1	4	[37]
*Burkholderia pseudomallei*	Case report/series	1	1	[86]
**Viruses**				
HTLV-1	Cross-sectional study in TL patients	3	2	[87–89]
HTLV-1	Cross-sectional study in HTLV-1–infected subjects	1	8	[90]

^a^Some overlap is possible because several papers come from the same research group.

Abbreviations: HTLV-1, human T-lymphotropic virus 1; TL, tegumentary leishmaniasis.

### Frequency of TL coinfections in human populations

The studies providing information about the frequency of coinfection in human populations are summarised below and in Table 1.

#### *Leishmania* and helminths

Two Brazilian cohort studies describe the frequency of helminth infections in patients with TL [5,12]. The first study recruited 120 patients with TL in a village health post in a rural area of Bahia state [5]. Only patients with cutaneous forms of leishmaniasis were included (maximum 4 lesions on maximum 2 body regions). The *Leishmania* species was not determined, but the predominant species in this region is known to be *L*. *braziliensis*. Study participants provided three stool samples for parasitological assays (sedimentation, Baermann, and Kato-Katz methods). Of the 120 patients with TL, 106 (88%) were diagnosed with a helminth infection. Seventy-three percent of the study participants were infected with more than one helminth species at the same time. The most common helminths in this study were *Ancylostoma duodenale*, *Trichuris trichiura*, *Ascaris lumbricoides*, *Schistosoma mansoni*, and *Strongyloides stercoralis*.

The second study was done in an urban area in the state of Rio de Janeiro [12]. This was a retrospective cohort study of 109 TL patients who received antimony therapy in a referral centre between 2004 and 2006: there were 99 cases of cutaneous and 10 of mucocutaneous leishmaniasis. All included patients had a parasitologically confirmed diagnosis of leishmaniasis. The species was typed in samples from 47 patients; they were all *L*. *braziliensis*. Parasitological examination of stool samples using sedimentation, Kato-Katz, and Baermann-Moraes methods was routinely performed during the study period. Sixteen (15%) out of 109 TL patients had helminth infections. The most frequent helminths were Ancylostomidae, *A*. *lumbricoides*, *S*. *stercoralis*, *S*. *mansoni*, and *T*. *trichiura* [12].

#### *Leishmania* and other Trypanosomatidae

The existence of coinfection with *T*. *cruzi* was proven in Argentina in 1996 [33]. Seven (58%) out of 12 patients with TL were diagnosed with *T*. *cruzi* infection based on specific serological tests. In three of the seven coinfected patients, the presence of *T*. *cruzi* could be proven with a direct parasitological technique (i.e., xenodiagnosis using *Triatoma infestans* nymphs). Six additional studies confirmed, based on specific serological and molecular techniques, that *T*. *cruzi* coinfection is frequent in TL patients from Salta in northern Argentina [31, 34–37,43], where the seroprevalence of *T*. *cruzi* in rural populations is estimated to range between 4% and 30% [31,91]. In all these studies, the coinfected patients had clinical TL but no signs of cardiac abnormalities typical of Chagas disease at the time of recruitment. The largest study included 330 patients with TL caused by *L*. *braziliensis* or *L*. *amazonensis* and found coinfection with *T*. *cruzi* in 135 (41%) of them [36].

Coinfection with *T*. *cruzi* has also been found in other Latin American countries [30,32,39,40]. One study in a hospital in Los Yungas in Bolivia recruited 28 patients with TL caused by *L*. *braziliensis* complex, *L*. *mexicana* complex, or both and obtained positive PCR results for *T*. *cruzi* in 22 (79%) [32]. In Paraguay, 8 (8%) out of 101 patients with clinical TL coming from the Caazapá and Alto Paraná departments were suspected of carrying *T*. *cruzi* [39].

The largest prevalence study was done in Brazil and reported on the frequency of coinfection of *L*. *braziliensis*, *L*. *infantum* (syn. *L*. *chagasi*), and *T*. *cruzi* in a sample of 1,100 apparently healthy people living in fast-growing villages in the outskirts of São Luiz City, the capital of Maranhão State [30]. Diagnosis of *Leishmania* and *Trypanosoma* infections was based on serology and molecular testing of blood samples. Forty-one subjects (4%) were diagnosed with *L*. *braziliensis* infection only, 35 (3%) with *T*. *cruzi* only, 50 (5%) with *L*. *chagasi* only, 17 (2%) had *L*. *braziliensis* together with *L*. *chagasi*, 7 (1%) had *L*. *chagasi* together with *T*. *cruzi*, and 11 (1%) had *L*. *braziliensis* together with *T*. *cruzi*. None of the study participants had signs of past or present TL, visceral leishmaniasis, or Chagas disease.

#### *Leishmania* and human T-lymphotropic virus 1

Three small studies in Colombia, Peru, and Iran reported a low frequency of human T-lymphotropic virus 1 (HTLV-1) infection in patients with TL. The number of study participants with TL ranged from 4 to 92, and the frequency of HTLV-1 infection ranged from 0% to 4% in subgroups with different forms of TL (subclinical or clinical, acute or chronic) [87–89]. A fourth study, from Mashhad in Iran, also failed to confirm a clear link between these two infections. These authors reported that 8 out of 100 HTLV-1–infected candidate blood donors mentioned a history of cutaneous leishmaniasis, which was not significantly different from the frequency reported by 100 HTLV-1–negative candidate blood donors [90].

#### *Leishmania* and other pathogens

One study from Salta in northern Argentina looked into several coinfections at the same time [37]. In a series of 93 patients with parasitologically confirmed cutaneous (*n* = 50) or mucocutaneous (*n* = 43) leishmaniasis, 37% had one or more coinfection, i.e., intestinal parasites (*n* = 2), *T*. *cruzi* (*n* = 25), *Toxoplasma gondii* (*n* = 2), *Paracoccidioides brasiliensis* (n = 2), *Coccidioides posadasii* (*n* = 1), *M*, *tuberculosis* (*n* = 3), and/or *Treponema pallidum* (*n* = 4). The authors described that the frequency of coinfections was higher in patients with mucosal forms of leishmaniasis than in those with cutaneous leishmaniasis [37].

Our search retrieved no studies on the frequency of other coinfecting pathogens in TL patients or the general population, although there were some case reports and series. Therefore, we can only report on the absolute number of human cases with coinfection mentioned in the literature. We found reports of 16 cases of concurrent coinfection of *Leishmania* with *T*. *gondii*, 4 with *Sporothrix schenckii*, 10 with *P*. *brasiliensis*, 1 with *Cryptococcus laurentii*, 9 with *M*. *tuberculosis*, 25 with *M*. *leprae*, 1 with *M*. *ulcerans*, and 1 with *Burkholderia pseudomallei* (Table 1).

### Interactions between *Leishmania* and other pathogens in animal models and human subjects

#### Types of interaction

**Fig 2:**
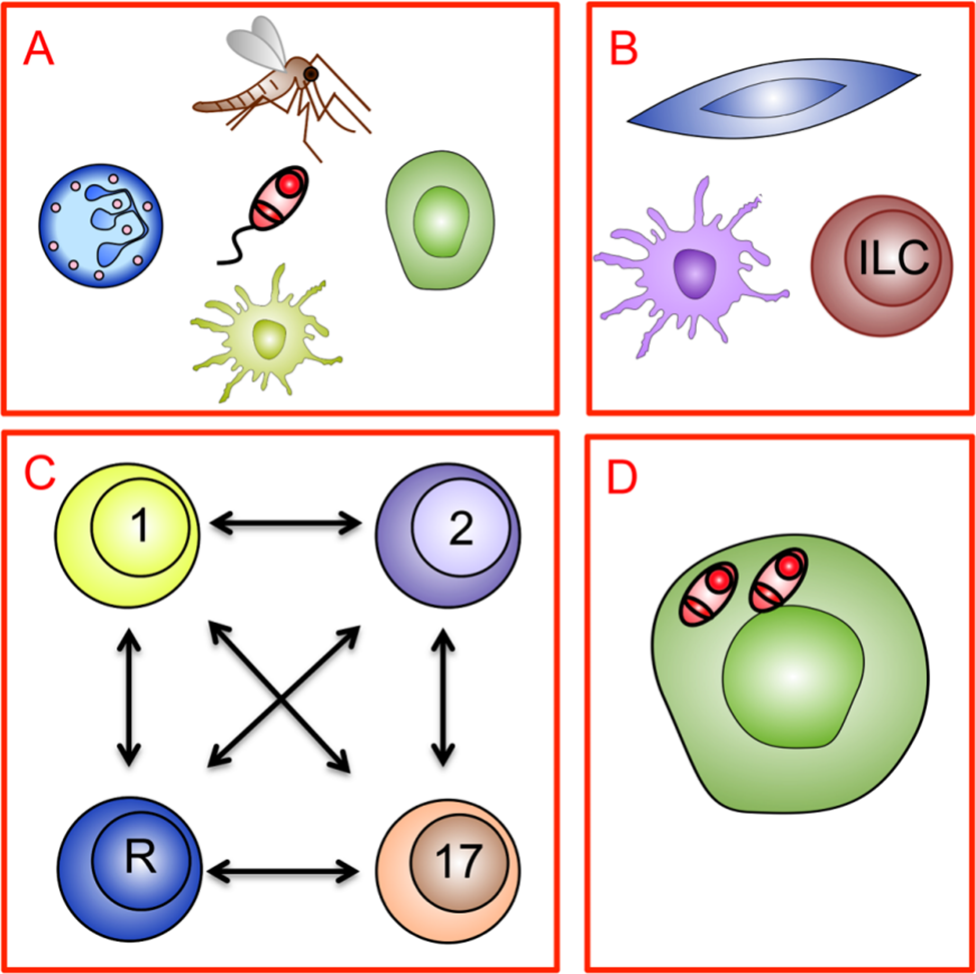
Immune responses during TL and the potential for interference through coinfection: A means to focus new research. (A) *Leishmania* parasite transmission during sandfly bite initiates TL. Local phagocyte function (including neutrophils, macrophages, and dendritic cells) may be affected by coinfections affecting skin homeostasis. Furthermore, coinfection may affect the nature of preexisting immunity to sandfly saliva and/or the local response to sandfly/parasite proteins. (B) Innate immune mechanisms regulated by stromal cells, dendritic cells, and innate lymphoid cells may all be influenced by the microenvironment created by local or systemic coinfection. (C) Changes to innate immunity or immunological cross-reactivity may influence the balance between effector (Th1, Th2, and Th17) and regulatory (R) T-cell subsets, leading to altered control of parasite load and/or altered immunopathology. (D) Coinfections may directly or indirectly alter macrophage intracellular signalling, affecting the intracellular survival of *Leishmania* independently of any effects on the specific T-cell response. ILC, innate lymphoid cell; Th, T helper cell; TL, tegumentary leishmaniasis.

Coinfections may influence the immune response during TL in several different ways: through actions on local phagocytes, innate immune mechanisms, the balance between effector and regulatory T-cell subsets, and the capacity of macrophages to kill *Leishmania* amastigotes (Fig 2).

There is considerable evidence supporting the roles of various key phagocyte populations (dermal macrophages, monocyte-derived macrophages and dendritic cells, and neutrophils) in the establishment of infection and first-line defence against *Leishmania* [92]. There is also a growing body of literature indicating that the functional attributes of these phagocytes can be influenced by products introduced during transmission (e.g., sandfly salivary proteins or parasite-derived immunomodulators) [93–95] or by changes in skin homeostasis (e.g., driven by pathologic coinfection or changes to the commensal microbiota) [96,97]. One study in mice showed that resident skin commensals were critical to promoting protective effector T-cell responses to *L*. *major* [98] and thus act as potent immunomodulatory coinfections necessary for the control of TL. However, specific publications about how phagocytes engaged in TL control may be affected by other pathogens or skin microbiota are currently lacking. Likewise, coinfection-associated changes in the function of innate lymphoid cells or mesenchymal stromal cells, although readily predicted from the literature, have yet to be shown to be relevant in established models of TL.

A well-known paradigm in immunity relates to the opposing effects of interferon-gamma (IFNγ) and interleukin-4 (IL-4) with regard to control of *L*. *major* lesion development in mice [99,100]. Whereas C57BL/6 mice self-heal under the control of IFNγ, BALB/c mice succumb to *Leishmania* infection in an IL-4–dependent manner. These counteracting cytokines were identified as the products of different subsets of CD4^+^ T helper (Th) cells (Th1 and Th2). The finding that these Th subsets/cytokines have different roles in the control of helminth versus *Leishmania* infection led to the notion that differing infections may skew T-cell immunity in polarised directions [100,101].

The included studies that contribute information about the interactions between *Leishmania* and specific other pathogens are summarised below per coinfecting agent. Most of these reports are based on research in animal models (*n* = 22), while only a few (*n* = 5) provide an extensive immunological characterisation of human coinfection. Most of the possible interaction mechanisms outlined in Fig 2 have not been covered yet by the specific literature about TL and coinfections included in this review.

#### Helminths

The effect of helminth coinfection on the course of TL has been studied in mice models [7,23–29] and described in human patients [5,12,22], with mixed findings. Some of the studies in mice concluded that in the presence of helminth infection, the time between experimental infection with *Leishmania* and development of skin lesions increased [26,27], while others found that this prepatent period decreased [23] or remained unchanged [28]. The conclusions were also divided about the size of the TL lesions, finding larger [7], smaller [27], or similar lesions [25,28] in mice with helminth coinfection. One study with extended follow-up (16 weeks) showed that the impact of helminth coinfection on lesion growth was time dependent [26]. These divergent findings may be partly due to the parasites used in the experiments (*S*. *mansoni* or *Litomosoides sigmodontis*, with *L*. *mexicana* or *L*. *major*) and the time between the two experimental infections [23,26,27].

When it comes to explaining the effects of helminth coinfection on the course of TL, one experimental study suggested that the Th2 responses induced by helminth infection had systemic effects that down-regulated the initial, local Th1 response to *Leishmania* [26]. In contrast, several other studies found that helminth infection did not interfere with the generation of *Leishmania*-specific Th1-type responses [24,25,27–29]. Furthermore, two groups used in vitro models to show that macrophages from helminth-infected mice were impaired in their ability to kill *Leishmania* [7,26]. Three studies in mice also evaluated whether TL altered the course of helminth infections, but no measurable effect was reported [24,26,28].

Two cohort studies in Brazil compared the characteristics of TL in patients with and without helminthiasis [5,12]. The studies were conducted in Rio de Janeiro and Bahia, where *L*. *braziliensis* is predominant and pentavalent antimony is the recommended treatment. The study in Bahia enrolled 120 patients with cutaneous forms of TL (including 106 [88%] with helminthiasis), and the study in Rio de Janeiro enrolled 109 patients with cutaneous and mucocutaneous forms of TL (including 16 [15%] with helminthiasis). The helminths detected were *A*. *duodenale*, *T*. *trichiura*, *A*. *lumbricoides*, *S*. *mansoni*, and *S*. *stercoralis*. Both studies reported that the time to heal under pentavalent antimony treatment was longer for patients with TL and helminth infection than for patients with TL only [5,12]. The study in Rio de Janeiro also found significant associations of helminth coinfection with mucosal leishmaniasis and poor response to treatment [12].

#### Trypanosoma

Four experimental studies (in mice or squirrel monkeys) and one observational study in humans addressed the effect of *Trypanosoma* coinfection (*Trypanosoma brucei* or *T*. *cruzi*) on TL [46–49]. Experimental Chagas disease did not protect against leishmaniasis and vice versa [46], although there were elements of immune cross-reactivity [47]. For the studies evaluating the impact of *Trypanosoma* on time until *Leishmania* lesion development [46–49], the main finding was a reduction in lesion growth rate in coinfected animals. In some cases, protection from ulceration was reported [46,48,49]. Normal lesion growth returned once the *Trypanosoma* infection was treated [48]. In one study in squirrel monkeys, *L*. *braziliensis* coinfection was shown to block the increase in QRS interval, i.e., the depolarisation time of the cardiac ventricles, which is normally associated with *T*. *cruzi* infection. This finding led the authors to suggest that prior infection with *Leishmania* parasites might provide some protection against Chagas-related cardiopathy [46]. One human immunological study focused on T-cell responses and showed that TL patients coinfected with *T*. *cruzi* had a higher T-cell differentiation profile than patients with TL only [44].

#### Toxoplasma

Experimental studies in mice suggest that toxoplasmosis affects the course of leishmaniasis and vice versa [51,52]. Albino mice that were infected first with *L*. *major* and 30 to 70 days later with *T*. *gondii* developed more severe forms of leishmaniasis than mice infected with *L*. *major* alone [51]. By contrast, the course of toxoplasmosis was more benign in coinfected mice than in those infected with *Toxoplasma* alone [51]. Another study showed a different type of interaction. Here, BALB/c mice were experimentally infected first with *T*. *gondii* and 5 days later with *L*. *major*. The acute toxoplasmosis induced a strong Th1 response, and the BALB/c mice that are normally susceptible to leishmaniasis developed a level of resistance comparable to that of C57BL/6 mice [52]. In human patients, such positive or negative interactions between toxoplasmosis and TL have not been reported yet, although one in vitro study found that *T*. *gondii*–specific T cells are recruited into *L*. *braziliensis* lesions and could influence TL pathogenesis locally [50].

#### Plasmodium

Seven experimental studies assessed *Plasmodium* coinfection and TL [53–59]. In coinfection models of *Plasmodium yoelii* or *Plasmodium berghei* together with *Leishmania enrietti*, *L*. *mexicana*, or *L*. *amazonensis* in hamsters, C57BL/6 mice, and BALB/c mice, the coinfected animals had larger lesions than the animals with *Leishmania* infection only. There was also an adverse effect of leishmaniasis on the course of malaria because coinfected animals had increased parasitaemia and mortality compared with animals with *Plasmodium* infection only [53–58]. These effects may vary according to the *Leishmania* species because one study of *P*. *yoelii* in BALB/c mice reported different findings for *L*. *amazonensis* and *L*. *braziliensis* [59].

#### Sporothrix

Coinfection with *Sporothrix* may occur when fungal spores are inoculated in a TL lesion. In Colombia, it was suggested that such inoculations occur when people lance their TL lesions using *Sporothrix*-contaminated thorns [60]. There is also a case report linking coinfection with *Sporothrix* to traumatic injury and TL reactivation (Koebner phenomenon) [61].

#### M. tuberculosis

We found 9 studies (8 case reports and 1 cross-sectional study) describing 12 human patients with concurrent tuberculosis and TL (Table 1). Five out of these 12 patients had mucosal forms of TL, and 4 had other, nonlocalised forms; the type of TL was not described in 3 patients. Results of leishmanin skin tests (arguably an in vivo correlate of Th1 responses) were available for 6 coinfected patients: 5 were positive or strongly positive. More detailed analyses of T-cell responses were not performed. Some authors hypothesised that an episode of tuberculosis can trigger reactivation of latent leishmaniasis [65,67–69]. Others suggested that an underlying immune defect could lead to the development of several infectious diseases at the same time [70]. This was based on the study of one patient who had lepromatous leprosy, several leishmaniasis lesions, and miliary tuberculosis and in whom a reduced responsiveness to IL-12 was found [70].

#### M. leprae

The search retrieved 12 case reports/series of human patients with concurrent leprosy and TL, but none of them contained evidence of a significant interaction between the two infections. Leprosy and TL are both caused by obligate intracellular organisms and involve a broad spectrum of clinical, histopathological, and immunological manifestations [6,70,73–83]. The paucibacillary/pauciparasitic type of disease (tuberculoid leprosy and localised cutaneous leishmaniasis) is at one pole of the spectrum and reflects effective T-cell immunity. At the other pole of the spectrum is the multibacillary/multiparasitic type of disease (lepromatous leprosy and diffuse cutaneous leishmaniasis), which occurs when the antigen-specific T-cell response is depressed [70,82–83].

We found descriptions of five patients with lepromatous leprosy and localised TL [74,75,77–79]. In one of these cases, a man with lepromatous leprosy and mucosal leishmaniasis, skin reaction and IFNγ production against *Leishmania* antigens were strong, whereas the responses against *M*. *leprae* antigens were almost absent [78,79]. Therefore, despite the similarities in the pathogenesis of TL and leprosy, patients can have a divergent T-cell response to each pathogen, indicating a degree of compartmentalisation of T-cell immunity. Nonetheless, follow-up of one patient suggested that IL-10–mediated regulatory responses induced during leprosy may help control the immunopathology of mucosal leishmaniasis [78,79]. Twenty other patients described in the literature had disease manifestations of leprosy and TL that were not that far apart on the disease spectrum [6,70,73,74,76,80–82].

In addition to these naturally occurring combinations of TL and leprosy, we found descriptions of artificially induced coinfection [83,84]. In the 1950s and 1960s, it was common practice in some *Leishmania*-endemic areas to immunise people against leishmaniasis by the inoculation of live *Leishmania tropica* parasites (‘leishmanisation’). Two papers report on the clinical and histopathological evolution of 24 Israeli patients with lepromatous leprosy who received a vaccination with living *Leishmania* parasites. Twenty-three patients showed the classical clinical progression of cutaneous leishmaniasis at the site of inoculation. The authors suggested that this clinical response to vaccination was similar to that of people without leprosy [83]. One additional patient with lepromatous leprosy, described in a separate report, developed diffuse leishmaniasis after vaccination, but (also in this person) the lesions healed spontaneously. These observations also suggest that leprosy does not alter the course of TL or vice versa [84].

### Implications of TL coinfections for clinical practice

#### Clinical similarities complicating diagnosis

A first diagnostic challenge occurs when there are clinical similarities between the lesions caused by *Leishmania* and some other pathogens. When one aetiological diagnosis is well established, a clinician may be tempted to attribute all the patient’s lesions to this one infection and stop examining the patient for symptoms and signs of other diseases. This may happen, for example, in patients with concurrent leprosy and leishmaniasis, particularly when patients have many skin lesions [82]. Furthermore, 2 case reports describe a year-long delay in the diagnosis of mucosal leishmaniasis because nasal symptoms were first attributed to leprosy [77,78]. Mucosal leishmaniasis can also be confused with mucosal manifestations of tuberculosis. Several authors have emphasised the importance of examining multiple samples from different skin lesions when coinfection is suspected [73–75,82]. Diagnosis of coinfection can become particularly challenging when more than one pathogen is present within the same lesion. *Leishmania* parasites have been found in skin or mucosal lesions together with *S*. *schenckii*, *C*. *laurentii*, *M*. *tuberculosis*, *M*. *leprae*, and *M*. *ulcerans* [6,60,61,64,65,85].

#### Biological similarities complicating diagnosis

A second diagnostic challenge stems from the biological similarities between *Leishmania* parasites and other pathogens. This problem is well documented for *Leishmania* and *T*. *cruzi*, which are both kinetoplastid protozoa with antigenic similarities. When conventional serological tests are used for the diagnosis of Chagas disease, there is a problem of cross-reactivity with *Leishmania*. There have been several attempts to develop serological tests that differentiate *Leishmania* from *T*.*cruzi* infections [38,39,41,42] and to evaluate their diagnostic performance in settings in which both pathogens are endemic [42,43]. Tests using purified or recombinant specific antigens of *T*. *cruzi*, such as Ag163B6, Ag162B6/cruzipain, or shed acute phase antigen (SAPA), proved to be useful to identify true coinfections [41,42].

#### Issues with the interpretation of diagnostic test results

One Brazilian study found that 52 out of 107 patients with a definite diagnosis of sporothrichosis also had one or more positive immunological test results for leishmaniasis (leishmanin skin test, ELISA, or indirect immunofluorescence test) [62]. The diagnosis of TL could not be confirmed in this study because parasitological confirmation tests were negative (*n* = 24) or not done (*n* = 28). It was therefore not possible to distinguish between true coinfections, serological cross-reactions, or false-positive results of the leishmanin skin test due to an allergy to the diluent [62]. The authors emphasise that in such a setting, incorrect diagnoses of TL are possible in patients with sporotrichosis and that, even in the presence of suggestive clinical and epidemiological arguments together with positive immunological test results for TL, parasitological confirmation is still needed before patients are exposed to a toxic and possibly unnecessary TL treatment [62].

#### Treatment sequence

The first therapeutic challenge in patients with coinfection is to determine the best sequence of the different treatments. Because helminth coinfection appears to increase the time to healing in patients with cutaneous leishmaniasis [5,12], it seems logical to assume that prompt diagnosis and treatment of helminth infections may improve the outcome of TL treatment. One randomised, double-blind, placebo-controlled trial in Bahia, Brazil, examined early versus deferred treatment of helminth coinfection [22]. This trial enrolled 90 patients with cutaneous leishmaniasis (most probably caused by *L*. *braziliensis*) and helminth coinfection (mainly hookworms, *T*. *trichiura*, *A*. *lumbricoides*, *S*. *mansoni*, and *S*. *stercoralis*). All participants were treated with intravenous antimony at 20 mg/kg/day for 20 days. The treatment group also received triple antihelminthic therapy with albendazole, ivermectin, and praziquantel at days 0 and 30 and placebo at day 60. The control group received placebo at days 0 and 30 and specific antihelminthic therapy based on stool test results on day 60. There was no significant difference between the two groups in the time to healing of the skin lesions: the median time to cure was 98 days in the treatment group and 88 days in the control group [22].

#### Treatment side effects

When two infections are treated at the same time, the drug combinations may lead to increased intolerance or adverse effects. The combination of antimony with antituberculous drugs is feared, and we found a description of death due to renal failure that was attributed to the combined treatment [67]. The combination treatment for TL (with pentavalent antimony) and leprosy (with diaminodiphenyl sulfone plus rifampicin plus clofazimine) may also produce considerable side effects [6]. Furthermore, several authors have raised concerns about the use of antimonial treatment for TL in patients with Chagas disease [40,45]. Pentavalent antimony drugs are known to prolong QT time and cause arrhythmia; they are therefore contraindicated in patients with known heart disease. On the one hand, cardiomyopathy is a well-known clinical manifestation of Chagas disease, and therefore prudence is called for in patients with *Leishmania*–*Trypanosoma* coinfection [40,45].

#### Unexpected responses to treatment

Some case reports discussed unexpected benefits of 1 treatment on 2 infections. For example, there was a report about a patient with chagasic cardiomyopathy and TL [45]. Amiodarone was used to control the patient’s ventricular arrhythmia and seemed to promote the healing of TL. The authors considered that amiodarone could have had an antileishmanial effect although they could not rule out the possibility that the use of amiodarone coincided with the healing of TL by chance [45].

Another interesting case was reported in Colombia [69]. A patient diagnosed with mucocutaneous leishmaniasis and pulmonary tuberculosis first received treatment for tuberculosis with rifampin, isoniazid, streptomycin, and pyrazinamide over a period of 7 months. The antimonial treatment was deferred because of concerns about the adverse effects of the combination of antituberculous and antimonial drugs. Despite the lack of specific antileishmanial treatment, when assessed 3 months after the end of antituberculous therapy, the mucosal lesions were fibrosed, scar tissue was evident, and the patient was biopsy culture negative. A similar observation was reported in Brazil, where the lesions of a patient with diffuse cutaneous leishmaniasis temporarily improved while the patient was receiving antituberculous therapy [66]. Some studies have suggested that streptomycin, isoniazid, and rifampin may have direct antileishmanial activity [66]. Alternatively, this response might reflect an interaction between TL and tuberculosis. For example, reduction of mycobacterial burden may release regulatory pressure within the immune system that also favours resolution of mucosal lesions, or antituberculous treatment may (re)activate host protective mycobacteria-specific T cells that cross-react with *Leishmania* antigens.

## Discussion

### Summary of main findings

This is the first comprehensive review of the literature about TL and coinfections other than HIV. Coinfection adds to the complexity of TL: the outcome of a single *Leishmania* infection in humans is difficult to predict, and the impact of coinfection on the course of TL is even more puzzling. Nevertheless, coinfection is clinically relevant because it is frequent, it can lead to diagnostic errors and delays, and it can influence the effectiveness of treatment and drug side effects. Therefore, it is crucial to gain a better understanding of the interaction between TL and other infectious diseases.

The frequency of coinfections has been studied mostly in Latin America so far. There is relatively good evidence about *T*. *cruzi* infection in Argentina (an estimated 41% of TL patients also carry *T*. *cruzi*) [36] and about helminthiasis in Brazil (an estimated 15% to 88% of TL patients also carry helminths) [5,12].

Several hypotheses have been explored about the mechanisms of interaction between the different microorganisms, but no clear answers have emerged so far from a literature that is scattered and still developing. Such interactions may involve one or all components of innate immunity coupled with the complexity of regulatory networks that affect the quality and quantity of the acquired immune responses (e.g., T-cell subset bias or regulatory cytokine production). Given that TL pathology is fundamentally an immunopathology reaction, coinfections could paradoxically lead to exacerbated TL disease by enhancing immune responses against *Leishmania* parasites in lesions. The impact of *Plasmodium* coinfection on TL in animal models is clearly detrimental; the impact of all other coinfections in animal models or human studies is less clear or less consistent.

Diagnostic problems occur when concurrent infections cause similar lesions (e.g., TL and leprosy), when different pathogens are present in the same lesions (e.g., *Leishmania* and *S*. *schenckii*), or when cross-reactions induced by phylogenetically close pathogens affect the accuracy of diagnostic tests (e.g., serology for leishmaniasis and Chagas disease). Regarding treatment, some coinfections seem to reduce the efficacy of antileishmanial drugs (i.e., helminthiasis), and there may be cumulative adverse effects caused by drugs or drug combinations (e.g., antimonial treatment in patients with chagasic cardiomyopathy, and combinations of antileishmanial and antimycobacterial drugs).

### Strengths and limitations

The strengths of this review are the broad search of the literature and the fact that the reporting follows PRISMA guidelines [21]. On the other hand, because the search strategy had few restrictions, we retrieved information in heterogeneous formats. As a consequence, we could not systematically assess the risk of bias in the individual records and decided to include all the available information. Most animal studies predate the introduction of the Animals in Research: Reporting *In Vivo* Experiments (ARRIVE) guidelines for reporting animal research [102]; therefore, issues related to experimental design and the avoidance of bias may not have been explicitly recorded in the publications reviewed.

Despite the broad search including several databases other than MEDLINE, the retrieved information was fragmented, and the evidence was insufficient to give firm answers to all the review questions. For example, all the evidence about TL and malaria came from animal studies without validation in humans. By contrast, all the information about tuberculosis came from human case reports with limited information about pathogenesis. In total, only 3 out of the 73 included records were cohort studies or clinical trials specifically designed to investigate the impact of coinfection on the course of TL in humans. Furthermore, there was not enough information available to look into the effect of coinfections on different clinical forms of TL (i.e., localised, diffuse, disseminated, and mucosal) separately. This is an important limitation because the host immune responses underlying these different forms of TL are contrasting and may be differentially modified by coinfections. For example, coinfections that induce a strong proinflammatory response could be beneficial in early cutaneous but detrimental in mucosal leishmaniasis. Finally, there was almost no information about coinfection in human subjects from Africa or Asia.

Several factors may have contributed to the lack of evidence about coinfections. First, coinfections tend to get less attention than single infections. Second, TL and many of the relevant coinfections are neglected diseases that affect poor populations and are typically under-researched and underreported. Finally, the complexity of TL together with other infections may lead to negative results or findings that are difficult to explain, which may reduce the chance of publication.

### Implications for future research

From a clinical point of view, several questions remain to be resolved. Even if the interactions between pathogens are complex, these clinical questions are fairly straightforward. For each of the coinfecting microorganisms, we need to better document (i) how frequent it is among patients with TL in different settings, (ii) whether TL patients with the coinfection fare better or worse than patients without it, (iii) whether the presence of the coinfection affects the accuracy of diagnostic tests, and (iv) what the best way to treat the coinfected patient is. With advances in the development of vaccines for leishmaniasis, including TL, an understanding of how vaccine responses might be modulated due to coinfection also becomes a question of some significance.

With regard to the interaction between pathogens, additional mechanisms, unexplored in the literature to date in relation to TL, are worthy of consideration. First, metabolic disturbances resulting from coinfection may alter the capacity of the immune system to appropriately respond during TL or vice versa [103,104]. Second, coinfections, in particular with helminths, may lead to a dysbiosis (i.e., alterations in the development or composition of the microbiota) that consequently impacts immune health [97,104,105]. Therefore, the answer to how the clinical outcome differs between single- and coinfected patients may not lie in understanding how two specific sets of immune responses interact but rather in how these responses are linked via complex regulatory circuits established and maintained by our commensal microbiota.

Several elements of the design of future experimental research deserve consideration. First, it is important to clarify what the outcomes of interest are, i.e., the risk of symptomatic disease, the time between infection and lesion appearance, the size of the lesion, time to healing, response to treatment, or risk of metastasis and comorbidities. The impact of coinfections on these different clinical outcomes may vary. Second, the species, the infective doses, and the timing of *Leishmania* and coinfection may also matter. Finally, animal models differ from each other, and they do not always represent what happens in human coinfection.

## Conclusion

In patients with TL, coinfection with other pathogens may be the rule rather than the exception. More research is needed to unravel how other infections interfere with the pathogenesis of TL. It is important that clinicians bear in mind the possibility of coinfection because this can complicate diagnosis and treatment.

## Supporting information

S1 List PRISMA checklist. PRISMA, Preferred Reporting Items for Systematic Reviews and Meta-Analyses.
(DOCX)

S1 File Command used to search MEDLINE via PubMed. (DOCX)
