# Supplementary material for: Tegumentary leishmaniasis and coinfections other than HIV
Source: PLoS Negl Trop Dis. 2018 Mar 1;12(3):e0006125. doi: 10.1371/journal.pntd.0006125 (PMC5832191; doi:10.1371/journal.pntd.0006125)
Supplement: S1 List — PRISMA, Preferred Reporting Items for Systematic Reviews and Meta-Analyses. (DOCX) [file pntd.0006125.s001.docx]

PRISMA checklist for the manuscript “Tegumentary leishmaniasis and coinfections other than HIV” by Martínez DY *et al*.

The checklist is taken from: Moher D, Liberati A, Tetzlaff J, Altman DG, The PRISMA Group (2009). Preferred Reporting Items for Systematic Reviews and Meta-Analyses: The PRISMA Statement. PLoS Med 6(7): e1000097. doi:10.1371/journal.pmed1000097. More information is available from www.prisma-statement.org.

The 27 PRISMA items are copied using *italic font*; the way in which we have addressed each of these items in our manuscript is described using regular, not-italic font.

1. *TITLE - Identify the report as a systematic review, meta-analysis, or both.*

We do not claim that this manuscript is a systematic review because our focus was broad (more than one review question) and because the available information was diverse (e.g. different types of coinfection and divergent study designs). Nevertheless, as described below, we took a systematic approach to searching literature, selecting records and obtaining information from the included records. The title of the manuscript is “Tegumentary leishmaniasis and coinfections other than HIV”. The fact that the manuscript is a review is mentioned early in the abstract.

1. *ABSTRACT - Provide a structured summary including, as applicable: background; objectives; data sources; study eligibility criteria, participants, and interventions; study appraisal and synthesis methods; results; limitations; conclusions and implications of key findings; systematic review registration number.*

Applicable elements are included in the abstract; the review protocol was not registered.

1. *INTRODUCTION - Describe the rationale for the review in the context of what is already known.*

People infected with *Leishmania* may carry other pathogens as well. These other pathogens may alter the host immune response against *Leishmania* infection and hence the clinical course of leishmaniasis. The interaction between tegumentary leishmaniasis and HIV is well established and has been reviewed before. This is the first comprehensive review of tegumentary leishmaniasis and coinfections with pathogens other than HIV.

1. *INTRODUCTION - Provide an explicit statement of questions being addressed with reference to participants, interventions, comparisons, outcomes, and study design (PICOS).*

The focus of this review is explained in the last paragraph of the introduction: “The objectives of the present review are to summarise the evidence about (i) the frequency of tegumentary leishmaniasis (TL) and coinfections other than HIV in human populations, (ii) interactions between *Leishmania* and other pathogens in animal models and human subjects, and (iii) implications of TL coinfections for clinical practice.”

1. *METHODS - Indicate if a review protocol exists, if and where it can be accessed (e.g., Web address), and, if available, provide registration information including registration number.*

No protocol has been registered for this review.

1. *METHODS - Specify study characteristics (e.g., PICOS, length of follow-up) and report characteristics (e.g., years considered, language, publication status) used as criteria for eligibility, giving rationale.*

We searched the medical literature to identify publications about TL and coinfections. To identify coinfections, we used search terms indicating (groups of) infections, pathogens, and diseases caused by these pathogens. For the purpose of this review, we defined TL as all forms of cutaneous (localised, disseminated or diffuse) and mucocutaneous leishmaniasis. Records about the skin manifestations caused by *L. donovani* and *L. infantum/L. chagasi* (such as post-kala-azar dermal leishmaniasis) were not included because the main clinical outcome of these infections is visceral leishmaniasis, which is outside the scope of this review. Records about HIV/AIDS and TL were not included because this topic has already been extensively reviewed elsewhere. Records about the contamination or superinfection of TL lesions with Gram-positive or Gram-negative bacteria of the skin such as *Staphylococcus aureus* or *Streptococcus pyogenes* were also excluded. Review papers were not included. We did not restrict the search by geographical region, study design, language of publication or publication date.

1. *METHODS - Describe all information sources (e.g., databases with dates of coverage, contact with study authors to identify additional studies) in the search and date last searched.*

Information for this review was identified up to August 2017 by searches of MEDLINE, Embase, LILACS, Scielo, Cochrane, African Index Medicus, as well as local library databases. We also reviewed the reference lists of selected articles.

1. *METHODS - Present full electronic search strategy for at least one database, including any limits used, such that it could be repeated.*

The detailed search strategy for MEDLINE is given in S1 File.

1. *METHODS - State the process for selecting studies (i.e., screening, eligibility, included in the systematic review, and, if applicable, included in the meta-analysis).*

One reviewer (DYM) screened titles and abstracts, and two reviewers (DYM and KV) assessed the eligibility of the full-text papers using the eligibility criteria outlined above (item 6). Doubts and discordances were resolved through discussion.

1. *METHODS - Describe method of data extraction from reports (e.g., piloted forms, independently, in duplicate) and any processes for obtaining and confirming data from investigators.*

Two reviewers (DYM and KV) read and summarised the included records. Doubts and discordances were resolved through discussion. We did not contact investigators to obtain additional information or to confirm data.

1. *METHODS - List and define all variables for which data were sought (e.g., PICOS, funding sources) and any assumptions and simplifications made.*

Specific points of interest while reading and summarising the articles were: (i) frequency of coinfection in humans; (ii) mechanisms of interaction and effect of coinfection on TL progression; and (iii) potential implications for clinical management.

1. *METHODS - Describe methods used for assessing risk of bias of individual studies (including specification of whether this was done at the study or outcome level), and how this information is to be used in any data synthesis.*

Our search did not include restrictions in study design and retrieved information in various formats. As a consequence, we did not formally assess the risk of bias of individual studies but described the different study designs instead.

1. *METHODS - State the principal summary measures (e.g., risk ratio, difference in means).*

The information was found in heterogeneous formats. We described the information the same way the authors of the original publications did, using counts, proportions and medians.

1. *METHODS - Describe the methods of handling data and combining results of studies, if done, including measures of consistency (e.g., I2) for each meta-analysis.*

This review does not include a meta-analysis.

1. *METHODS - Specify any assessment of risk of bias that may affect the cumulative evidence (e.g., publication bias, selective reporting within studies).*

Not done

1. *METHODS - Describe methods of additional analyses (e.g., sensitivity or subgroup analyses, meta-regression), if done, indicating which were pre-specified.*

Not done

1. *RESULTS - Give numbers of studies screened, assessed for eligibility, and included in the review, with reasons for exclusions at each stage, ideally with a flow diagram.*

The MEDLINE search retrieved 669 records and searching other databases yielded 348 additional records. After reading titles or abstracts or both, we removed 79 duplicates and discarded 841 records because they were not relevant (Fig 1). The most frequent reason for dropping records was that while leishmaniasis and another infection were mentioned in the same text, the publication was not about coinfection (e.g. a paper about different infections occurring in the same region but not affecting the same persons). We assessed the remaining 97 full-text records for eligibility and retained 73 for the present review (Fig 1).

1. *RESULTS - For each study, present characteristics for which data were extracted (e.g., study size, PICOS, follow-up period) and provide the citations.*

Table 1 gives an overview of all the included studies. This table describes according to the coinfecting pathogen and the study design: the number of included studies, the number of human cases with coinfection, and the citations.

1. *RESULTS - Present data on risk of bias of each study and, if available, any outcome level assessment (see item 12).*

Study design is described instead of risk of bias: the 73 articles included in this review had different study designs. There were 21 original research papers about experimental studies of coinfection in animal models, and 52 original research papers about coinfection in human patients. The 52 studies about human subjects included 1 clinical trial, 2 cohort studies, 13 cross-sectional or prevalence studies, 7 studies on the development or performance of diagnostic tests, 24 case series or case reports with a clinical focus, and 5 case series or reports with an immunological focus.

1. *RESULTS - For all outcomes considered (benefits or harms), present, for each study: (a) simple summary data for each intervention group (b) effect estimates and confidence intervals, ideally with a forest plot.*

Main findings are summarised following a different structure: frequency of TL coinfections in human populations, interactions between *Leishmania* and other pathogens, and implications of TL coinfections for clinical practice.

1. *RESULTS - Present results of each meta-analysis done, including confidence intervals and measures of consistency.*

Not done

1. *RESULTS - Present results of any assessment of risk of bias across studies (see Item 15).*

Not done

1. *RESULTS - Give results of additional analyses, if done (e.g., sensitivity or subgroup analyses, meta-regression [see Item 16]).*

Not done

1. *DISCUSSION - Summarize the main findings including the strength of evidence for each main outcome; consider their relevance to key groups (e.g., healthcare providers, users, and policy makers).*

The discussion contains a specific section entitled ‘summary of main findings’.

1. *DISCUSSION - Discuss limitations at study and outcome level (e.g., risk of bias), and at review-level (e.g., incomplete retrieval of identified research, reporting bias).*

The discussion contains a specific section entitled ‘strengths and limitations’.

1. *DISCUSSION - Provide a general interpretation of the results in the context of other evidence, and implications for future research.*

The discussion contains a specific section entitled ‘implications for future research’.

1. *FUNDING - Describe sources of funding for the systematic review and other support (e.g., supply of data); role of funders for the systematic review.*

DYM received a PhD scholarship from the Belgian Directorate General for Development Cooperation (third framework agreement, project 95502). The funders had no role in study design, data collection and analysis, decision to publish, or preparation of the manuscript.
