# Supplementary material for: Tegumentary leishmaniasis and coinfections other than HIV
Source: PLoS Negl Trop Dis. 2018 Mar 1;12(3):e0006125. doi: 10.1371/journal.pntd.0006125 (PMC5832191; doi:10.1371/journal.pntd.0006125)
Supplement: S1 File — (DOCX) [file pntd.0006125.s002.docx]

**MEDLINE search strategy via PubMed**

**Nr Search terms**

#1 (leishmani*) OR oriental sore

#2 post kala-azar dermal leishmaniasis

#3 1 NOT 2

#4 (((visceral) ANDleishmani*) OR "Leishmaniasis, Visceral"[Mesh]) OR “Kala-azar”

#5 3 NOT 4

#6 ((coinfect*) OR co-infect*)

#7 superinfect*

#8 (coexist*) OR co-exist*

#9 ((((concur*) OR concomit*) OR associat*) OR mix*) OR second*

#10 infect

#11(8 OR 9) AND 10

#12 complicat*

#13 ((6 OR 7) OR 11) OR 12

#14 (HIV) OR AIDS

#15 13 NOT 14

#16 5 AND 15

#17 (malaria) OR plasmodium

#18 mycobacterium

#19 (((leprosy) OR hansen*) OR tuberculos*) OR Buruli*

#20 ((trypanosom*) OR Chagas) OR sleeping sickness

#21 sporotri*

#22 ((helminth*) OR schistosom*) OR strongyloid*

#23 toxoplasm*

#24 HTLV

#25 ((((((17 OR 18) OR 19) OR 20) OR 21) OR 22) OR 23) OR 24

#26 5 AND 25

#27 16 OR 26
